# Analysis of xanthyletin and secondary metabolites from *Pseudomonas stutzeri* ST1302 and *Klebsiella pneumoniae* ST2501 against *Pythium insidiosum*

**DOI:** 10.1186/s12866-019-1452-4

**Published:** 2019-04-16

**Authors:** Kittiya Wittayapipath, Saline Laolit, Chavi Yenjai, Sirinart Chio-Srichan, Maitree Pakarasang, Ratree Tavichakorntrakool, Chularut Prariyachatigul

**Affiliations:** 1 Department of Clinical Microbiology, Faculty of Associated Medical Sciences, Centre for Research and Development of Medical Diagnosis Laboratories, Khon Kaen University, Khon Kaen, 40002 Thailand; 2 Department of Chemistry, Faculty of Science, Khon Kaen University, Khon Kaen, 40002 Thailand; 3 Synchrotron Light Research Institute (Public Organization), Nakhon Ratchasima, 30000 Thailand

**Keywords:** *P. insidiosum*, *P. stutzeri* ST1302, *K. pneumoniae* ST2501, Xanthyletin, Synchrotron radiation-based Fourier-transform infrared (FTIR) microspectroscopy

## Abstract

**Background:**

*Pythium insidiosum* is a member of the oomycetes class of aquatic fungus-like microorganisms. It can infect humans and animals through skin wounds and the eyes, causing pythiosis, an infectious disease with high morbidity and mortality rates. Antifungal agents are ineffective as pythiosis treatments because ergosterol, the target site of most antifungal agents, is not found in the *P. insidiosum* cytoplasmic membrane. The best choice for treatment is surgical removal of the infected organ. While natural plant products or secretory substances from bacterial flora have exhibited in vitro anti-*P. insidiosum* activity, their mechanism of action remains unknown. Therefore, this study hypothesized that the mechanism of action could be related to changes in *P. insidiosum* biochemical composition (such as lipid, carbohydrate, protein or nucleic acid) following exposure to the inhibitory substances. The biochemical composition of *P. insidiosum* was investigated by Synchrotron radiation-based Fourier-transform infrared (FTIR) microspectroscopy.

**Results:**

Fraction No.6 from the crude extract of *P. stutzeri* ST1302, fraction No.1 from the crude extract of *K. pneumoniae* ST2501 and xanthyletin were used as anti-*P. insidiosum* substances, with MFCs at 3.125, 1.57–1.91, 0.003 mg/ml, respectively. The synchrotron FTIR results show that the deconvoluted peak distributions in the amide I, amide II, and mixed regions were significantly different between the treatment and control groups.

**Conclusions:**

Xanthyletin and the secondary metabolites from *P. stutzeri* ST1302 and *K. pneumoniae* ST2501 exerted anti-*P. insidiosum* activity that clearly changed the proteins in *P. insidiosum*. Further study, including proteomics analysis and in vivo susceptibility testing, should be undertaken to develop a better understanding of the mechanism of anti-*P. insidiosum* activity.

## Background

*Pythium insidiosum* is a member of the oomycete class of aquatic fungus-like microorganisms. It can infect both humans and animals through the eyes or skin wounds to cause pythiosis, a disease with severe morbidity and high mortality rates [1, 2]. Hosts become infected after biflagellate zoospores that found in wetland areas attach to a skin wound [1, 3, 4]. The clinical manifestations are comprised of cutaneous and subcutaneous forms, vascular form, ocular form, and disseminated pythiosis. The treatment options that have been employed for pythiosis include antifungal agents, radical surgery, and immunotherapy. However, these treatments have not been successful in all cases [1, 5]. Most antifungal agents are ineffective because *P. insidiosum* lacks ergosterol in the cytoplasmic membrane, the target site of antifungal agents, which act by interfering with ergosterol synthesis and its function in the membrane [1]. The most effective treatment protocol for both humans and animals with pythiosis is radical surgery, but patients can suffer from serious disabilities [2, 6]. Interestingly, the biological control works by reducing the numbers of pests or eliminating pest organisms by interfering with their ecological status through the introduction a natural competition or pathogen into the environment. Natural plant products and secretory substances from bacterial flora have been reported to have in vitro anti-*P. insidiosum* activity. A new furanocoumarin isolated from the fruit of *Scaevola taccada* and secondary metabolites from *Pseudomonas stutzeri* and *Klebsiella pneumoniae* that were collected from swampy areas have also been found to exhibit in vitro inhibitory effects on the human pathogenic oomycete *P. insidiosum* [7, 8]. Nonetheless, the precise mechanism of this inhibitory effect remains unknown.

Synchrotron Fourier-transform infrared (FTIR) microspectroscopy utilizes infrared (IR) radiation from a synchrotron light source that is transmitted through an interferometer to irradiate a sample and cause biomolecular vibrations. Biomolecules have unique vibrational characteristics which correspond to specific infrared light frequencies that are related to their functional groups [9–11]. FTIR spectra can provide an informative semi-quantitative overview of the lipid, protein, carbohydrate or nucleic acid components of a biological sample. This technique is a proven and accepted tool for the study of biological samples. The present study therefore used synchrotron FTIR microspectroscopy to study the biochemical composition changes in *P. insidiosum* after the organism was exposed to inhibitory substances, based on the presumption that these changes would reflect the mechanism of its inhibitory activity.

## Results

### Crude extraction from *P. stutzeri* ST1302 and *K. pneumoniae* ST2501

The crude extracts from *P. stutzeri* ST1302 and *K. pneumoniae* ST2501 were divided into 14 and 5 fractions, respectively. Fraction No.6 (yellow solid) of the crude extract from *P. stutzeri* ST1302 was eluted with 20% methanol in dichloromethane, while fraction No.1 (brown semisolid) of the crude extract from *K. pneumoniae* ST2501 was eluted with 100% dichloromethane gave the strongest anti-*P. insidiosum* activity. Both the crude extract and fraction No.1 from the crude extract of *K. pneumoniae* ST2501 gave stronger anti-*P. insidiosum* activity and stability results than *P. stutzeri* ST1302.

### Anti-*P. insidiosum* activity and minimum fungicidal concentration

Xanthyletin and the crude extracts from *P. stutzeri* ST1302 and *K. pneumoniae* ST2501 showed anti-*P. insidiosum* activity against all eleven of the clinical strains of *P. insidiosum* by disc diffusion method. The MFCs were 3.125, 1.57–1.91 and 0.003 mg/ml for fraction No.6 from the crude extract of *P. stutzeri* ST1302, fraction No.1 from the crude extract of *K. pneumoniae* ST2501, and xanthyletin, respectively (Table 1).

**Table 1:** The minimal fungicidal concentrations (MFCs) of fraction No.6 from crude extract of *P. stutzeri* ST1302, fraction No.1 from crude extract of *K. pneumoniae* ST2501, and xanthyletin (mg/ml)

*Pythium insidiosum* strains	MFC of fraction No.6 of crude extract from *P. stutzeri* ST1302 (mg/ml)	MFC of fraction No.1 of crude extract from *K. pneumoniae* ST2501 (mg/ml)	MFC of xanthyletin (mg/ml)
*P. insidiosum* SIMI-6666	3.125	1.910	0.003
*P. insidiosum* SIMI-2989-42	3.125	1.740	0.003
*P. insidiosum* SIMI-7873	3.125	1.740	0.003
*P. insidiosum* SIMI-7874	3.125	1.740	0.003
*P. insidiosum* MCC29	3.125	1.570	0.003
*P. insidiosum* MCC5	3.125	1.570	0.003
*P. insidiosum* SIMI-18093	3.125	1.570	0.003
*P. insidiosum* SIMI-322-37	3.125	1.570	0.003
*P. insidiosum* SIMI-8659	3.125	1.570	0.003
*P. insidiosum* SIMI-8727	3.125	1.570	0.003
*P. insidiosum* SIMI-9743	3.125	1.570	0.003

### Synchrotron radiation-based FTIR microspectroscopy

**Fig. 1:**
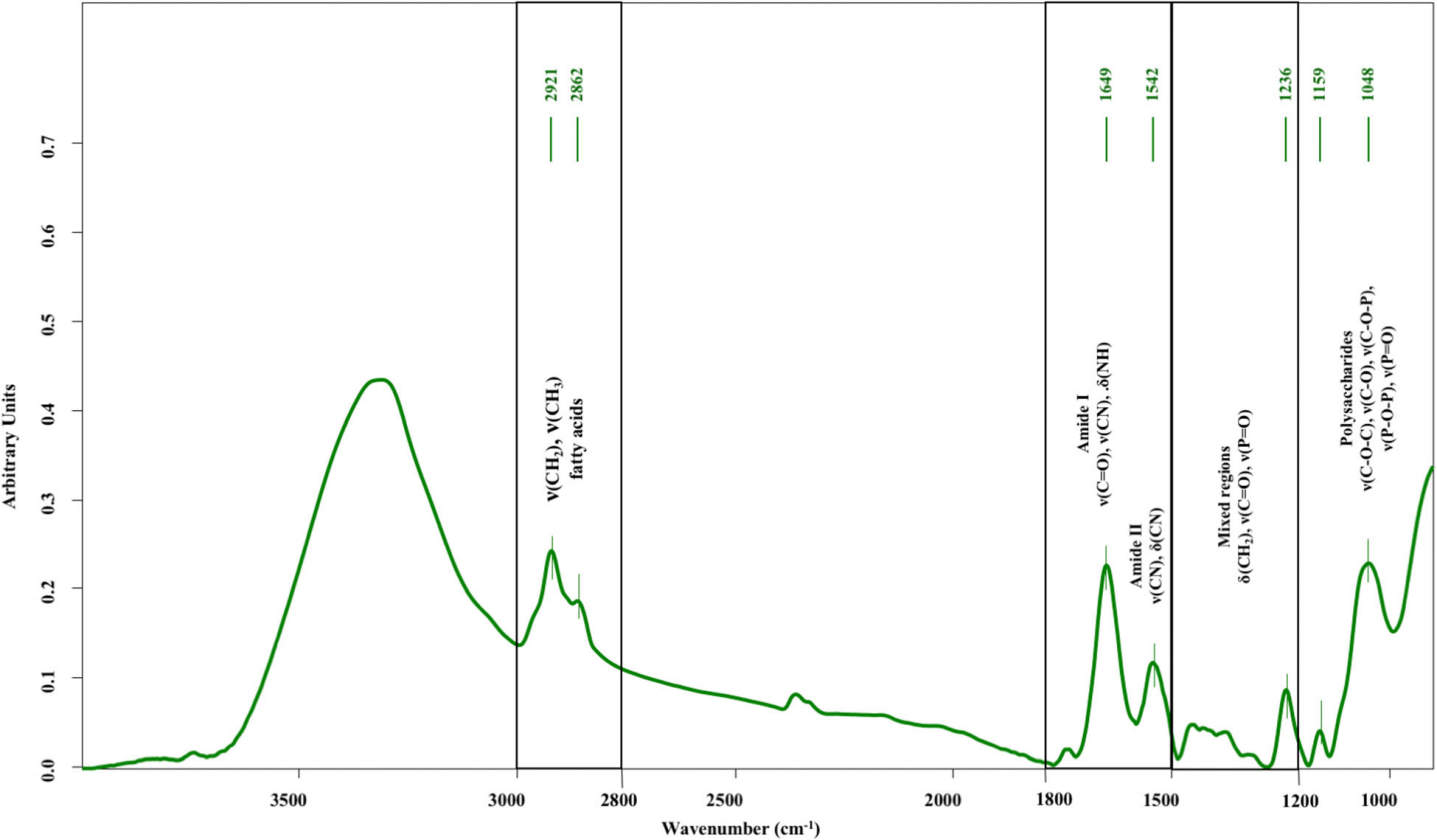
Characteristic spectrum of *P. insidiosum*. The different molecular bonds are indicated with their biomolecular attribution (ν = stretching vibration, δ = bending vibration)

**Fig. 2:**
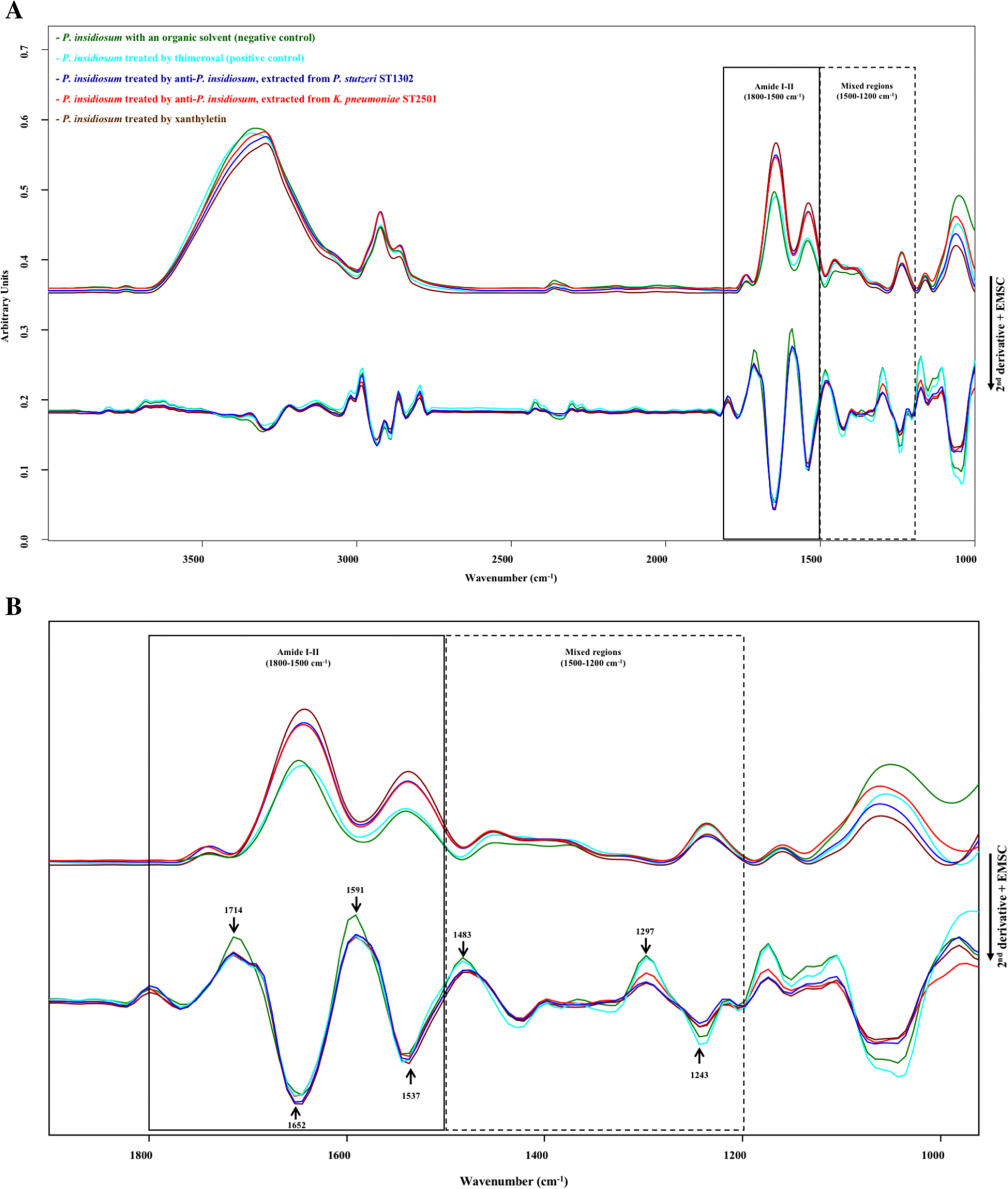
The average spectra of *P. insidiosum* with treated and control groups. **a** The raw average spectra model (upper lines) derived from *P. insidiosum* with an organic solvent as the negative control (green), *P. insidiosum* treated by thimerosal as the positive control (sky blue) and *P. insidiosum* with the three differences treated by xanthyletin (carmine) and anti-*P. insidiosum* substances that extracted from *P. stutzeri* ST1302 (navy) and *K. pneumoniae* ST2501 (red) showed the pattern of the whole spectrum (4000–900 cm^− 1^). All the average spectra from the second derivative and EMSC processing (lower lines) demonstrated the different regions in the spectral range of 1800–1500 cm^− 1^ (protein region) and 1500–1200 cm^− 1^ (mixed region). **b** Enlarging image at the average spectral range 1900–900 cm^− 1^ revealed clearly differences points in proteins and mixed regions of the spectra which managed by the second derivative and EMSC, whereas the raw average spectra were not seen

The spectral profile provided information about the biochemical composition and corresponded with important macromolecules such as the proteins, lipids, and carbohydrates of filamentous *P. insidiosum* (Fig. 1). Firstly, the region between 3000 and 2800 cm^− 1^ demonstrates the C-H stretching vibrations of CH_3_ and CH_2_ functional groups in fatty acid chains of the diverse membrane, such as phospholipids. The next region, from 1800 to 1500 cm^− 1^, shows the stretching vibrations of C=O, CN and bending mode of NH in amide I and the C-N stretching vibrations and bending mode in amide II. Subsequently, the region between 1500 and 1200 cm^− 1^ which represents a mixed region that exhibited C=O symmetric stretching vibrations of -COO^−^ functional groups of amino acid side chains or free fatty acids, CH_2_ bending mode of lipids and proteins, and P=O asymmetric stretching vibrations in the head group of phospholipids. Meanwhile, the region between 1200 and 900 cm^− 1^ is demonstrated the symmetric stretching vibration of PO_2_^−^ groups in nucleic acids and C-O-C, C-O and C-O-P stretching vibrations of various oligo and polysaccharides [12–14]. The spectral analysis showed that the spectral windows from 1800-1500 cm^− 1^ and 1500–1200 cm^− 1^ were the regions with the greatest number of alteration effects on the different treatments and control groups of *P. insidiosum* (Fig. 2).

Fig. 2 reveals the raw spectra (upper lines) and the spectra from the second derivative and EMSC processing (lower lines), derived from the average spectra of the whole spectral range (3000–900 cm^− 1^) of the 5 groups (Negative control, Positive control, *P. insidiosum* treated by anti-*P. insidiosum* that extracted from *P. stutzeri* ST1302, *P. insidiosum* treated by anti-*P. insidiosum* that extracted from *K. pneumoniae* ST2501, and *P. insidiosum* treated by xanthyletin). The average second derivative and EMSC spectra showed an amide I peak at 1652 cm^− 1^, 1537 cm^− 1^ of amide II and P=O stretching vibration of phospholipids at 1243 cm^− 1^. Moreover, the spectral windows from 1800 to 1200 cm^− 1^ exhibited seven peaks, including at 1714, 1652, 1591, 1537, 1483, 1297 and 1243 cm^− 1^ which are different points in the five spectra groups.

### Chemometric analysis

**Fig. 3:**
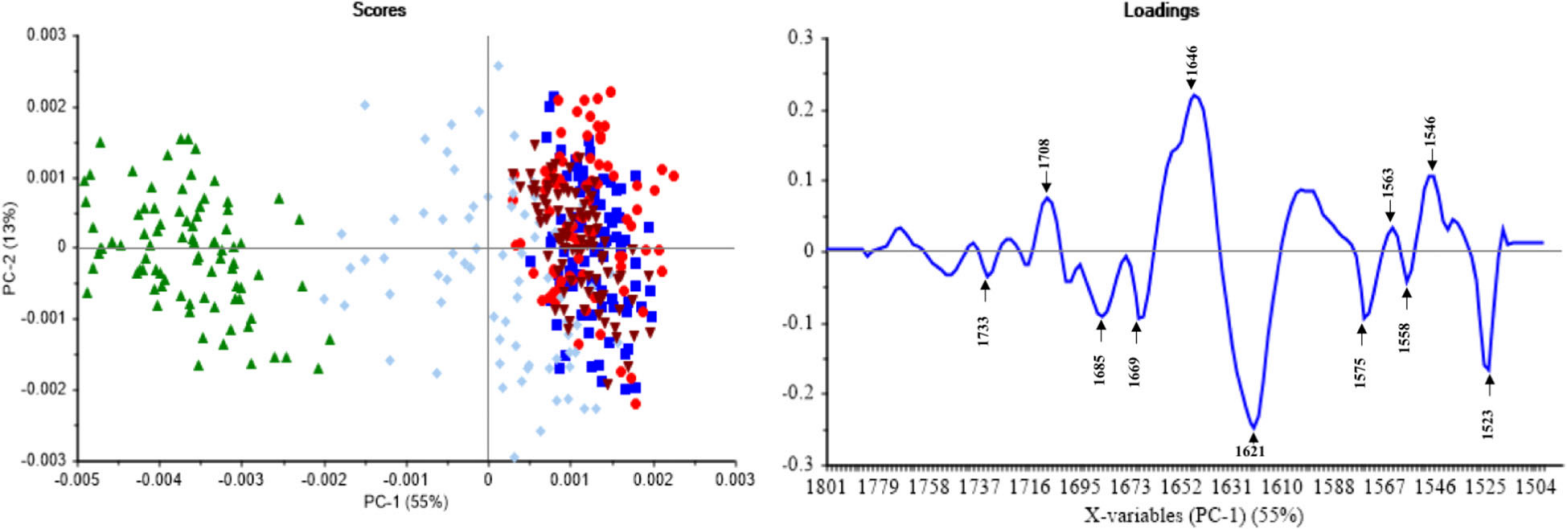
The PC scores plot and loadings plot from the PCA of 1800–1500 cm^− 1^. ▲ *P. insidiosum* with an organic solvent (negative control), ♦ *P. insidiosum* treated by thimerosal (positive control), ■ *P. insidiosum* treated by anti-*P. insidiosum* that extracted from *P. stutzeri* ST1302, ● *P. insidiosum* treated by anti-*P. insidiosum* that extracted from *K. pneumoniae* ST2501, and ▼ *P. insidiosum* treated by xanthyletin

**Fig. 4:**
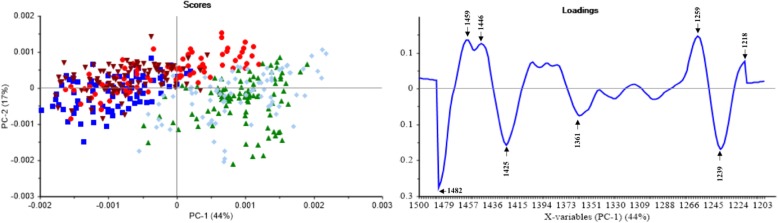
The PC scores plot and loadings plot from the PCA of 1500–1200 cm^− 1^. ▲ *P. insidiosum* with an organic solvent (negative control), cc *P. insidiosum* treated by thimerosal (positive control), ■ *P. insidiosum* treated by anti-*P. insidiosum* that extracted from *P. stutzeri* ST1302, ● *P. insidiosum* treated by anti-*P. insidiosum* that extracted from *K. pneumoniae* ST2501, and ▼ *P. insidiosum* treated by xanthyletin

PCA was performed in four regions; 3000–2800 cm^− 1^ as a fatty acid region corresponding to lipid membrane, 1800–1500 cm^− 1^ as an amide I and II region corresponding to proteins and peptides, 1500–1200 cm^− 1^ as a mixed region containing vibrations of fatty acids, proteins, and polysaccharide, and 1200–900 cm^− 1^ as a polysaccharide region corresponding to carbohydrates. Each sample showed absorption bands in all four areas. We found significant differences between the treated and the control groups, in both the amide I and amide II region (Fig. 3) as well as in the mixed region (Fig. 4).

Fig. 3 shows the PC scores plot and loadings plot obtained from the second derivative and EMSC spectra in the amide I and amide II region (1800–1500 cm^− 1^). The two dimensional of PC scores plot (PC1 and PC2) showed 68% of the total variance. The PC1 (55%) was reliable for the separation of *P. insidiosum* from the treatment groups, the positive control and the negative control. The PC2 (13%) explains the variation within each group. The loadings plot revealed a significant peak shift at the amide I (1646 cm^− 1^ shift to 1621 cm^− 1^) and amide II (1546 cm^− 1^ shift to 1523 cm^− 1^), representing the effect of thimerosal and anti-*P. insidiosum* on the amide I and amide II structure of *P. insidiosum*.

Fig. 4 shows the PC scores plot and loadings plot obtained from the second derivative and EMSC spectra in the mixed region (1500–1200 cm^− 1^). The two dimensional of PC scores plot (PC1 and PC2) showed 61% of the total variance. The PC1 (44%) was responsible for the separation of *P. insidiosum* from the treatment and control groups, in which the positive and negative controls were distributed in the same area and were separated from treatment groups. The PC2 (17%) explains the variation within each group. The loading plot revealed a significant peak shift at the CH_2_ bending mode of lipids and proteins (1482 cm^− 1^ shift to 1459 cm^− 1^) and P=O asymmetric stretching vibrations in the head group of phospholipids (1239 cm^− 1^ shift to 1218 cm^− 1^).

**Fig. 5:**
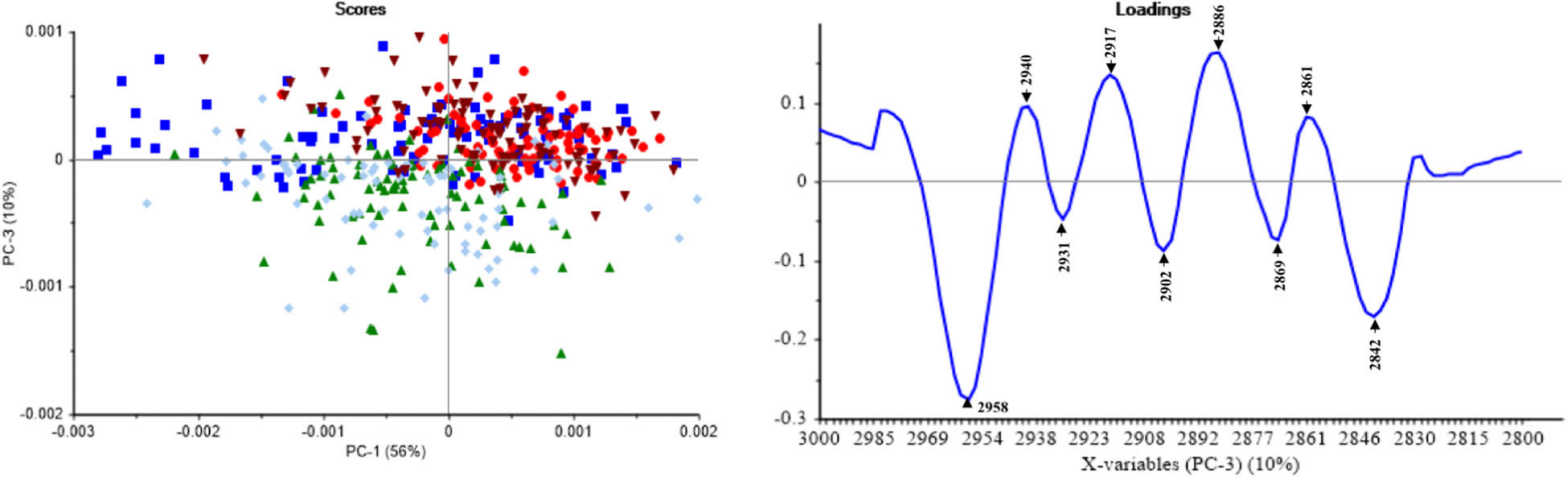
The PC scores plot and loadings plot from the PCA of 3000–2800 cm^− 1^. ▲ *P. insidiosum* with an organic solvent (negative control), ▲ *P. insidiosum* treated by thimerosal (positive control), ■ *P. insidiosum* treated by anti-*P. insidiosum* that extracted from *P. stutzeri* ST1302, ● *P. insidiosum* treated by anti-*P. insidiosum* that extracted from *K. pneumoniae* ST2501, and ▼ *P. insidiosum* treated by xanthyletin

Fig. 5 shows the PC scores plot and loadings plot obtained from the second derivative and EMSC spectra in the fatty acid region (3000–2800 cm^− 1^). The two dimensional of PC scores plot (PC1 and PC3) showed 66% of the total variance. The PC3 was reliable for the separation of *P. insidiosum* from the treatment and control groups, in which the positive and negative controls were distributed in the same area and were slightly separated from the treatment groups. However, PC3 explained 10% of the separation of the sample groups, while PC1 showed 56% of the variation within each group. The PCA from the second derivative and EMSC spectra in the polysaccharides region (1200–900 cm^− 1^) couldn’t separate in any of the sample groups [13–15].

The PCA results of the four regions show that the protein components were changed in all the groups that were treated with anti-*P. insidiosum* substances and the positive control, whereas the mixed regions containing lipids, proteins and phospholipids were changed in the three groups which were treated with xanthyletin and secondary metabolites from bacteria. No significant differences were found in the fatty acid and polysaccharide regions in all the sample groups.

## Discussion

Antagonistic microorganisms play a major role in microbial equilibrium and serve as powerful agents for biological disease control [16]. *Pseudomonas stutzeri* has been used to control fungal diseases in plants incited by *Fusalium solani, Fusarium oxysporum, Fusarium moniliformae, Fusarium udum, Macrophomena phaseolina, Rhizoctonia solani, Colletotrichum capsicii, Aspergillus flavus,* and *Aspergillus niger* [17]. Moreover, *Klebsiella pneumoniae* exhibits anti-fungal activity through secreted bioactive compounds that suppress the growth of *Aspergillus flavus, Aspergillus fumigatus, Candida albicans, Penicillium expansum, Aspergillus niger* and *Aspergillus terreus*. Abeer et al. reported that 100 mg/mL of crude extract from *K. pneumoniae* gave higher diameter zones of inhibition than 30 μg/mL of amphotericin B, fluconazol and miconazole nitrate when used against *Aspergillus flavus* [18]. In the present study, crude extracts and fractions from *K. pneumoniae* ST2501 gave stronger anti-*P. insidiosum* activity and stability than the crude extracts and fractions from *P. stutzeri* ST1302. This was due to *K. pneumoniae* ST2501 producing many more important secondary metabolites with high biological activities when compared to *P. stutzeri* ST1302. Abeer Fauzi Al-Rubaye et al. [18], reported that *K. pneumoniae* produces many important secondary metabolites with high biological activities as anti-fungal activity.

Coumarins are members of a class of compounds called benzopyrones, which consist of fused benzene and α-pyrone rings. Most coumarins are secondary metabolites of green plants, but some coumarins are produced by fungi and bacteria. Xanthyletin is a member of *pyranocoumarins* (coumarin derivatives) and has been shown to have anti-fungal activity against *Candida albicans* and filamentous fungi [19]*.* Of the tested compounds in the present study, xanthyletin had the strongest anti-*P. insidiosum* activity. The observed MFC of 0.003 mg/ml correlated well with compound 5 from the fruits of *Scaevola taccada* that showed anti-*P. insidiosum* activity with the MIC of 0.005 mg/ml [7]. A higher potential for xanthyletin activity against *P. insidiosum* hyphae is promising for the development of antimicrobial drugs against pythiosis.

Synchrotron radiation-based FTIR microspectroscopy was used to for the identification of filamentous fungi along with analyzed spectra data and statistical analysis of biomolecular discrimination [9, 10]. This is the first study of *P. insidiosum* hyphae by synchrotron radiation-based FTIR microspectroscopy that shows the spectral data specific to functional groups of biological molecules such as proteins, lipids, and carbohydrates. Statistical analysis of the FTIR spectra found protein changes in *P. insidiosum* when treated using thimerosal (positive control), the three sample groups treated with xanthyletin, and two anti-*P. insidiosum* substances from *P. stutzeri* ST1302 and *K. pneumoniae* ST2501. However, the pattern of the proteins changes in the positive control and the three treated sample groups were different. Thimerosal’s anti-microbial action is dependent on it breaking down and releasing ethyl-mercury to penetrate the cell membranes and binds to intracellular enzymes, which then inhibits them and causes cell death [20]. Interestingly, previous studies have shown that some anti-bacterial drugs, such as aminoglycosides, tetracyclines and macrolides, exhibit inhibitory activity against *P. insidiosum*, but the MICs are incompatible with safe plasma levels [21, 22]. While the mechanism of action of these anti-bacterial drugs against eukaryotic organisms is not entirely understood, their mechanisms are similar to be similar to that described for *P. ultimum*: reduced incorporation of amino acids into proteins, inhibition of protein synthesis, and inhibition of amino acid transport [23]. Therefore, we propose that the target molecule of the xanthyletin and the secondary metabolites from *P. stutzeri* ST1302 and *K. pneumoniae* ST2501 might be protein components of this organism.

## Conclusion

In vitro studies of xanthyletin and the secondary metabolites from *P. stutzeri* ST1302 and *K. pneumoniae* ST2501 showed anti-*P. insidiosum* activity, characterized by clearly changed proteins in *P. insidiosum*. Future study should seek to include proteomics analysis to improve understanding about the mechanism of anti-*P. insidiosum* activity and an in vivo study of these anti-*P. insidiosum* extracts.

## Methods

### Microorganisms preparation

*Pythium insidiosum* preparation: Eleven clinical strains of *P. insidiosum*, isolated from the ocular, vascular, and disseminated forms of human pythiosis were identified by Assoc. Prof. Dr. Angkana Chaiprasert, Department of Microbiology, Mahidol University. These organisms were rechecked by culture on Sabouraud Dextrose Agar at 25 °C, detect perpendicular sparsely septate hyphae under the light microscope, induced zoospore formation and PCR identification [8]. The cultures were maintained on Sabouraud Dextrose Agar at room temperature and sub-cultured once a month. This study was approved by the Center for Ethics in Human Research, Khon Kaen University (number HE592105).

*Pseudomonas stutzeri* ST1302 and *Klebsiella pneumoniae* ST2501 preparation. *P. stutzeri* ST1302 and *K. pneumoniae* ST2501 that produced anti-*P. insidiosum* substances were isolated from water sampling area around Khon Kaen University and identified by Dr. Yordhathai Thongsri [8]. The microorganisms were rechecked by the automated Vitex2 system and preserved in skimmed milk with 15% glycerol and then frozen at − 20 °C.

Xanthyletin: Commercially xanthyletin (C_14_H_12_O_3_) powder as a coumarin compound was obtained from ChemFaces (Wuhan, China). It showed 98% of purity tested and 228.24 of molecular weight.

### Crude extraction from *P. stutzeri* ST1302 and *K. pneumoniae* ST2501

The *P. stutzeri* ST1302 and *K. pneumoniae* ST2501 inoculants were prepared to McFarland Standard No.1 (3 × 10^8^ CFU/ml), 700 μl of this concentration was inoculated by aseptic technique into six 1000 ml bottles, each of which contained 700 ml of brain heart infusion (BHI) broth. Each bottle was then incubated on a rotary shaker at 200 rpm at 37 °C for 3 days. Then, 4.2 l of culture broth was centrifuged at 7500 rpm for 20 min at 4 °C and sterilized by filtration through a 0.45 μm pore size membrane filter (Millipore). Cell free filtrate was concentrated ten-fold in a rotary evaporator (Rotavapor R-210, Buchi). Four hundred and twenty milliliter of the cultured broth was then mixed three times with a two-fold volume of dichloromethane (Fisher) for *P. stutzeri* ST1302, and ethyl acetate (Fisher) for *K. pneumoniae* ST2501. Each dichloromethane and ethyl acetate layer was then dried with anhydrous Na_2_SO_4_ (Merck) and concentrated in a rotary evaporator (Rotavapor R-210, Buchi).

### Separation of the crude extracts from *P. stutzeri* ST1302 and *K. pneumoniae* ST2501

The crude extracts from *P. stutzeri* ST1302 and *K. pneumoniae* ST2501 were fractionated by activity-guided separation liquid column chromatography (LCC). The extracts were applied to a glass column filled with a slurry of silica gel 60 (0.015–0.040 mm; Merck), which had been preconditioned with dichloromethane for the crude extract from *P. stutzeri* ST1302, and with ethyl acetate for the crude extract from *K. pneumoniae* ST2501. Gradient mixtures of dichloromethane and methanol were used as mobile phases. The eluent was collected and fractions defined according to their thin layer chromatography (TLC) characteristics. TLC was using silica gel GF254 pre-coated aluminum sheets. The fractions containing the same major compounds were combined and tested for anti-*P. insidiosum* activity using the disc diffusion method described in the following section. The fraction with inhibitory activity from *P. stutzeri* ST1302 was separated again using LCC with gradient mixtures of dichloromethane and hexane (Merck) as mobile phases. TLC was then used for the differentiation of single fractions according to retention factors (R_f_) and to check purity. Detection was under exposure to UV light at 256 and 364 nm.

### Anti-*P. insidiosum* activity by disc diffusion method

Testing solutions, including the whole crude extract, fractions of the crude extract and xanthyletin, were tested for anti-*P. insidiosum* activity. The testing solutions were prepared as 500 mg/ml stock solutions. Paper discs (6 mm in diameter; Gibthai, Thailand) were placed onto SDA plates with *P. insidiosum* and grown for 2 days, and 20 μl of each testing solution was added to the discs (dichloromethane and ethyl acetate were used as a negative controls). The testing plates were stored at room temperature for 2 h in a laminar flow biosafety cabinet to testing the solution diffusion and they were then incubated at 37 °C for 3, 6 and 9 days. Inhibition zones were measured when the growth of *P. insidiosum* reached the negative control discs.

### Broth dilution susceptibility testing

To investigate the in vitro anti-*P. insidiosum* effect of the crude extract from the bacteria and xanthyletin, we used the method of Rodrigo Trolezi et al. with some modifications [24]. Briefly, fragments of *P. insidiosum* mycelia (5 mm in diameter) from the SDA plates were inoculated by aseptic technique into 1.95 ml of Sabouraud Dextrose Broth (SDB) and incubated at 37 °C for 3 days. Fifty microliters of varying concentrations of the testing solutions that had anti-*P. insidiosum* activity were added to the cultures and incubated at 37 °C for 24 h. Fifty microliters of organic solvent, which dissolved the anti-*P. insidiosum* substances, was added and incubated at 37 °C for 24 h as the negative control. The *P. insidiosum* fragments were then placed on SDA plates and incubated at 37 °C for 3, 6 and 9 days to follow the hyphal growth and determine the minimal fungicidal concentration (MFC). All tests were performed in triplicate.

### Sample preparation for FTIR microspectroscopy

According to the broth dilution susceptibility testing, the MFC of each testing solution was selected for FTIR microspectroscopy analysis. *P. insidiosum* treated by 0.02% (*w*/*v*) thimerosal was selected as the positive control, while *P. insidiosum* mycelia fragments in SDB plus organic solvent was selected as the negative control. After incubation, 1 ml of individual mycelia were collected and transferred to an Eppendorf tube and centrifuged (430 x g for 30 s) to remove the culture supernatant. The mycelia were then washed 3 times by 1 ml of 0.9% NaCl by centrifugation (430 x g for 30 s) and the supernatant was discarded. Mycelial pellets were resuspended in 300 ml of 0.9% NaCl before FTIR microspectroscopy analysis.

### FTIR-spectral acquisition and pre-processing

Five microliters of mycelia suspension were placed on a calcium fluoride (CaF_2_) window for synchrotron radiation-based Fourier transform infrared (FTIR) microspectroscopy investigation. The CaF_2_ windows were dried in a class II biological safety cabinet (BSC) for 30 min and kept in the desiccator until FTIR investigations to avoid the influence of water absorption on the infrared spectra. FTIR spectra were acquired using a Hyperion 2000 IR microscope coupling with a Vertex 70 spectrometer (BRUKER Optics GmbH, Ettlingen, Germany). The internal light source was replaced by synchrotron light delivered from the front-end of Beamline 4.1: IR at Synchrotron Light Research Institute (Thailand). The spectra were recorded in transmission mode using a 15x objective, 64 accumulations per sample, a spectral resolution of 4 cm^− 1^, and a spectral range of 4000–900 cm^− 1^. Before the samples were measured, the background spectrum of the CaF_2_ window was recorded and then subtracted from the sample signal. Pre-processing of infrared spectra included the region 4000–900 cm^− 1^, all spectral data were extracted by OPUS software (version 7.2, Bruker Optics GmbH, Ettlingen, Germany).

### Chemometric analysis

Principal Component Analysis (PCA) was performed using Unscrambler software (version 10.5, CAMO software AS, Norway) to classify the samples using their explained variables in the following spectral ranges; 3000–2800 cm^− 1^ as the fatty acid region (corresponding to lipid membrane), 1800–1500 cm^− 1^ as the amide I and II region (corresponding to proteins and peptides), 1500–1200 cm^− 1^ as the mixed region containing vibrations of free fatty acids, proteins, and phospholipids, and 1200–900 cm^− 1^ as the polysaccharide region (corresponding to carbohydrates). The second derivative was performed with Savitzky Golay 19-point smoothing which removed the broad baseline offset. In addition, the final spectra were taken as an extended multiplicative scatter correction (EMSC), which eliminated the physical information collected from the light scattering and removed artefacts that were introduced from normalizing the path length differences. This ensured that the spectra obtained would only be from the functional groups of the biomolecules. The EMSC corrected data is a robust representation of the biomolecules present. After the EMSC processing, the samples were classified using principal component analysis (PCA).

## Abbreviations

FTIR: Fourier transform infrared
MFC: Minimal fungicidal concentration
PC: Principal component
PCA: Principal component analysis
SDA: Sabouraud dextrose agar
SDB: Sabouraud dextrose broth
